# Transient Receptor Potential Vanilloid 1 (Trpv1) Receptors Are Expressed by Parietal Cells in the Rat Stomach Fundus and Antrum

**DOI:** 10.21203/rs.3.rs-10362099/v1

**Published:** 2026-07-22

**Authors:** Shyamal Premaratne, Hansa Doppalapudi, Nuveena S. Sooriya, Morgan B. Edwards, Mitchell L. Schubert

**Affiliations:** Virgina Union University; George Washington University; Maggie L. Walker Governor’s School for Government and International Studies; Virgina Union University; Medical College of Virginia at Virginia Commonwealth University

**Keywords:** Vanilloid, Primers, Gene Sequencing

## Abstract

We describe a simple technique using in-house-designed forward and reverse primers to detect the receptors for Transient Receptor Potential Vanilloid 1 (TRPV-1) in the fundus and antrum of the rat stomach. The use of RT-PCR, agarose gel electrophoresis, DNA band purification, sequencing, and, lastly, GeneBlast confirmed the presence of these receptors.

## INTRODUCTION

The endocannabinoid system (ECS) is a complex signaling network that is integral to regulating gastrointestinal homeostasis, influencing inflammation, digestive motility, and secretory functions throughout the gastrointestinal tract (1, 2). Among the receptors influenced by endocannabinoids is the transient receptor potential vanilloid type 1 (TRPV1 or VR1), a non-selective cation channel activated by heat, capsaicin, and certain lipid mediators (3). TRPV1 is expressed in various components of the gastrointestinal tract, particularly in gastric tissues, where it plays a critical role in sensory signaling, mucosal protection, and inflammatory response modulation (4).

In addition to their established role in gastrointestinal physiology, VR1 receptors (now more commonly known as TRPV1 or Transient Receptor Potential Vanilloid 1) are significantly linked to the cardiovascular system (5). TRPV1 is extensively expressed in the heart, blood vessels, and sensory nerves that innervate cardiovascular tissues. Through its expression in these structures, TRPV1 contributes to blood pressure regulation, ischemic responses, cardiac remodeling, and arrhythmogenesis (6). Activation of TRPV1 exerts cardioprotective effects during ischemic injury, while dysregulation of TRPV1 signaling has been correlated to cardiac hypertrophy, cardiac failure, and ventricular arrhythmias (7). These findings highlight the broader physiological significance of TRPV1 and suggest that it may serve as a promising therapeutic target across multiple organ systems.

Although TRPV1 has been detected in various gastric cell types, its specific localization within parietal cells remains unclear (8). This study aims to detect TRPV1 receptor expression in rat stomach tissues, specifically the fundus and antrum, by utilizing a molecular approach involving RT-PCR with customdesigned primers. Confirmation through sequencing supports the molecular identity of TRPV1 transcripts, contributing to our understanding of cannabinoid effects on gastric function. Determining whether these receptors are expressed in parietal cells could provide insight into novel mechanisms of acid regulation and neuroendocrine signaling in the stomach. Furthermore, understanding this localization may help enhance TRPV1’s potential as a therapeutic target in gastrointestinal disorders (9).

## EXPERIMENTAL PROCEDURES

### Animals

Male Sprague-Dawley rats weighing 150–200 g were deprived of solid food overnight but were allowed to drink water containing 10% dextrose. The animals were anesthetized with 20% urethan (5 ml/kg body weight, i.p.). All experimental protocols were approved by the Virginia Commonwealth University Institutional Animal Care and Use Committee.

### Tissue Collection

Gastric tissues, including the fundus and antrum, were harvested from adult male rats under appropriate ethical guidelines. The tissues were immediately frozen in liquid nitrogen and stored at −80°C until further processing.

### Parietal Cells Isolation

Parietal cells were then isolated from the gastric fundic and antral mucosa using previously described methods used in our laboratory (10).

### RNA Extraction and cDNA Synthesis

Total RNA was extracted from the isolated parietal cell preparations using a standard TRIzol-based method. RNA concentration and purity were assessed using spectrophotometry. Reverse transcription was performed using 1 μg of total RNA and a cDNA synthesis kit, yielding complementary DNA (cDNA) for subsequent amplification (10).

### Primer Design and PCR Amplification

PCR amplification targeted the TRPV1 receptor gene using uniquely designed primers:

- Upstream Primer: 5’-GACATGCCACCCAGCAGG-3'
- Downstream Primer: 5’-TCAATTCCCACACACCTCCC-3'

PCR was carried out in a 25 μL reaction volume using standard cycling parameters optimized for the TRPV1 gene. Amplification products were resolved on a 1.5% agarose gel to confirm the expected fragment size.

### Cloning and Sequencing

PCR products were subcloned into pGEM-T vectors and transformed into competent E. coli cells. Positive colonies were selected, and plasmids were extracted and sequenced to confirm the identity of the amplified TRPV1 receptor gene fragment.

## RESULTS AND DISCUSSION

RT-PCR using rat VTRPV1-specific primers yielded a band at the expected size for rat TRPV1 (987-bp) from both antral and fundic mucosa. The PCR product was cloned, and sequence analysis revealed complete homology with the known TRPV1 sequence.

**Figure 1:**
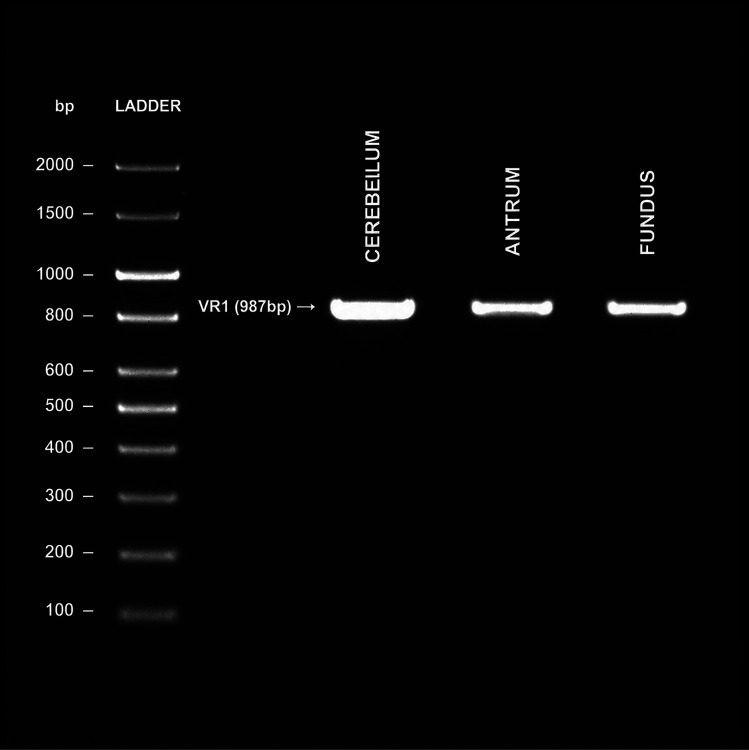
RT-PCR Analysis of TRPV1 Expression in Rat Gastric Fundus and Antrum

The successful amplification and sequencing of TRPV1 receptor transcripts from both fundic and antral regions of the rat stomach confirm the presence of these receptors in these gastric tissues (Fig. 1). The use of unique primers ensured specificity in detecting the target gene, minimizing off-target amplification. The subcloning and sequencing steps provided robust validation of the PCR results, affirming that the observed products corresponded to TRPV1 receptor cDNA.

This detection has significant physiological implications. The localization of TRPV1 receptors in the gastric mucosa supports the hypothesis that cannabinoids directly influence gastrointestinal function. One possible pathway is through the modulation of somatostatin, a key inhibitory hormone involved in the regulation of gastric secretions and motility. The molecular evidence provided here lays the groundwork for future functional studies investigating the specific roles of cannabinoid signaling in gastric hormone dynamics and mucosal health.
